# Development of a health education intervention strategy using an implementation research method to control taeniasis and cysticercosis in Burkina Faso

**DOI:** 10.1186/s40249-017-0308-0

**Published:** 2017-06-01

**Authors:** Helena Ngowi, Ivan Ozbolt, Athanase Millogo, Veronique Dermauw, Télesphore Somé, Paul Spicer, Lori L. Jervis, Rasmané Ganaba, Sarah Gabriel, Pierre Dorny, Hélène Carabin

**Affiliations:** 1 Department of Veterinary Medicine and Public Health, Sokoine University of Agriculture, P.O. Box 3021, Morogoro, Tanzania; 2 Department of Anthropology, University of Oklahoma, Norman, OK USA; 3 Language Department, Citizen Potawatomi Nation, Shawnee, OK USA; 4 Sourou Sanou University Teaching Hospital, Bobo-Dioulasso, Burkina Faso; 5 Department of Biomedical Sciences, Institute of Tropical Medicine, Antwerp, Belgium; 6 Agriculutral and Research Training Agency for Africa (AFRICSanté), Bobo-Dioulasso, Burkina Faso; 7 Department of Anthropology and Center for Applied Social Research, University of Oklahoma, Oklahoma City, OK USA; 8 Department of Biostatistics and Epidemiology, College of Public Health, Oklahoma University Health Sciences Center, Oklahoma City, OK USA

**Keywords:** *Taenia solium* control plan, Taeniasis, Cysticercosis, PHAST, Burkina Faso

## Abstract

**Background:**

Taeniasis and cysticercosis are two diseases caused by *Taenia solium*, a parasite transmitted between humans and pigs, leading to considerable economic loss and disabilities. Transmission of the parasite is linked to environmental and behavioural factors such as inadequate sanitation and hygiene, poor pig management, and consumption of infected pork. This study used implementation research method to design a health education intervention strategy for reducing *T. solium* infections in Burkina Faso, a country endemic for the parasite.

**Methods:**

Eighteen group discussions were conducted with 8–18 participants each in three villages. In addition, structured interviews were conducted among 4 777 participants and 2 244 pig owners, who were selected through cluster random sampling in 60 villages of three provinces of Burkina Faso. Both approaches assessed knowledge and practices related to *T. solium*. The information obtained was used to develop a community-adapted health education intervention strategy to control taeniasis and cysticercosis in Burkina Faso.

**Results:**

The group discussions revealed that participants had a poor quality of life due to the diseases as well as inadequate access to latrines, safe water, and healthcare services. In addition, it was found that pig production was an important economic activity, especially for women. Furthermore, financial and knowledge constraints were important limitations to improved pig management and latrine construction. The survey data also showed that open defecation and drinking unboiled water were common behaviours, enhanced by a lack of knowledge regarding the transmission of the parasite, perceived financial barriers to the implementation of control measures, lack of public sensitization, as well as a lack of self-efficacy towards control of the parasite. Nevertheless, the perceived financial benefits of controlling porcine cysticercosis could be emphasized by an education program that discourages open defecation and encourages drinking safe water. The final intervention strategy included a Participatory Hygiene and Sanitation Transformation (PHAST) approach, as well as a 52-min film and an accompanying comic booklet.

**Conclusions:**

The main problem in the study communities regarding the transmission of *T. solium* cysticercosis is the random disposal of human faeces, which can be contaminated with parasite eggs. Prevention of open defecation requires the building of latrines, which can be quite problematic in economically challenged settings. Providing the community with the skills to construct durable latrines using low-cost locally available materials would likely help to resolve this problem. Further studies are required to implement and evaluate the *T. solium* control strategy developed in this study.

**Electronic supplementary material:**

The online version of this article (doi:10.1186/s40249-017-0308-0) contains supplementary material, which is available to authorized users.

## Multilingual abstract

Please see Additional file 1 for translations of abstract into six official working languages of the United Nations.

## Background

In Burkina Faso, pigs serve as a common food source, being a relatively economical source of animal protein due to the pigs’ ability to obtain nutrition by scavenging on local refuse [1]. The country ranks second in terms of pig population in West Africa (following Nigeria) [2, 3]. *Taenia solium* is a parasite transmitted between humans and pigs causing cysticercosis, larval infection which can lead to serious neurological manifestations (including epileptic seizures) in humans [4]. Taeniasis, infection with the adult parasite in the small intestine in human can lead to gastrointestinal disturbances and it is the source of larval infections in humans and pigs. Transmission of *T. solium* is linked with practices such as open defecation, allowing pigs to roam freely, and poor access to safe water, which can put humans and pigs at risk of ingesting *T. solium* eggs from human faeces infected with the adult worm. Changing health-related behaviours and practices requires a context-specific analysis to identify important and potentially modifiable factors that can be targeted when developing strategies to effectively control the parasite.

Research has shown that human epilepsy and neurocysticercosis are important health problems in Burkina Faso, with neurocysticercosis being the cause of epilepsy in at least some areas of the country. In Burkina Faso, in 1990s 1.9% (*n* = 532) of seizure cases were considered probable cases of neurocysticercosis, and cysticercosis larvae were found in 0.5% (*n* = 3 410) of biopsy and surgical samples in two studies conducted at the university hospital of Ouagadougou [5, 6]. In a more recent cross-sectional study conducted in three villages of Burkina Faso, 0% (95% confidence interval, 95% *CI*: 0–2.1%) of those sampled in the village of Nyonyogo (*n* = 176), where there were very few pigs, had positive antigen enzyme-linked immunosorbent assay (Ag-ELISA) [7] test responses. In contrast, in the Pabré village (*n* = 288), where most pigs were confined, the seropositivity was 1.4% (95% *CI*: 0.4–3.5%), while in Batondo village (*n* = 302), where most pigs were left to roam, the seropositivity was 10.3% (95% *CI*: 7.1–14.3%) [8]. Among the 328 pigs examined, the Ag-ELISA-based prevalence of current porcine cysticercosis was estimated at 32.7% (95% Bayesian credible interval, BCI: 12.1–54.3%) in Batondo and 48.3% (95% BCI: 27.9–74.8%) in Pabré [9]. The prevalence of lifetime epilepsy was estimated to be 4.5% (95% *CI:* 3.3–6.0%) among the 888 sampled villagers [10], and the proportion of neurocysticercosis among people with epilepsy, diagnosed using computerized tomography, was estimated to be 46.9 and 45.5% in the two villages where pigs were raised. No neurocysticercosis cases were found in the village with very few pigs [10].

Health education includes consciously constructed opportunities for learning, involving some form of communication, designed to improve health literacy and develop life skills that are conducive to individual and community health [11]. Research has shown that effective health education interventions are based on a clear understanding of the targeted behaviours and the context in which they occur [12]. One of the essential elements of health education programs is the involvement of the community in all phases of program development from its planning to its implementation to its evaluation [12].

The Predisposing, Reinforcing, and Enabling Constructs in Educational Diagnosis and Evaluation (PRECEDE) model [13] is a planning model based on the premise that just as medical diagnosis precedes a medical plan so should an educational diagnosis precede an intervention plan. The original model follows five steps to guide the design of appropriate interventions based on theories of human health behaviour. The phases are elaborated elsewhere [13] and in the results section of this paper, and they include (1) Social Assessment, (2) Epidemiological Assessment, (3) Behavioural and Environmental Assessment, (4) Educational and Organizational Assessment, and (5) Administrative and Policy Assessment.

This model has been used successfully in the past in various public health settings. A study in Seattle, USA and Vancouver, Canada found that Chinese cultural beliefs play an important role in the dietary practices of Chinese people living in North America, and should thus be incorporated into the design and implementation of culturally appropriate health promotion programs for Chinese immigrants [14]. In Tanzania, the model was successfully used to develop culturally sensitive counselling tools for HIV and infant feeding [15] and effective health education strategies to reduce porcine cysticercosis [16].

This paper describes how an intervention strategy to control *T. solium* in Burkina Faso was developed using the PRECEDE model.

## Methods

### Study area

This study was conducted in three provinces of Burkina Faso located to the west of the capital city, Ouagadougou. Boulkiemdé and Sanguié were selected because they had the largest pig population in the country in 2010, with 191 438 and 145 923 pigs, respectively [17]. The third province, Nayala, was selected because it borders the other two provinces and also had a sizeable pig population (41 521 pigs). The 2006 national census reported that 505 206, 297 036, and 163 433 people were living in Boulkiemdé, Sanguié, and Nayala, respectively [18].

### Study design and data collection

This cross-sectional study used both qualitative and quantitative approaches to data collection. Group discussions (GDs) were conducted to understand community perceptions related to taeniasis and porcine cysticercosis and transmission risk factors in order to guide better plan of the questionnaire survey and ultimately the intervention to control the diseases. The GDs were conducted in three purposively villages selected to represent villages in the three selected provinces. A close-ended questionnaire, used to quantify knowledge and practices related to *T. solium* transmission, was completed by residents of 60 villages located in the three provinces of interest but different from those where the GDs took place.

#### Group discussions

As part of the ‘Social Assessment’ phase of the PRECEDE approach, six GDs were conducted in each of the three selected villages: Batondo (Sanguié), Kikigogo (Boulkiemdé), and Sawa (Nayala). These represented typical villages where pigs are raised in each study province. The GDs were comprised of separate groups of men (*n* = 9) and women (*n* = 9) including youths, adults, and older adults selected purposively to have a representation of the different age groups of the community. A total of 235 individuals participated in the GDs. The GD participants were primarily illiterate and mostly engaged in farming activities.

The GDs were conducted in December 2010 to assess community members’ perceptions of their quality of life in general; community-level sanitation (e.g. defecation, sources of drinking water); pig production and health; as well as willingness to implement disease control measures, with special attention paid to *T. solium* taeniasis and cysticercosis. The GDs were held at the market place in Batondo, at the village chief’s house in Kikigogo, and in three different settings in Sawa, sites that were found to be convenient by the study participants. In all villages, investigators worked with trained interpreters to conduct the GDs. The interpreters translated the answers from the languages Gurunsi, Mooré (Mossi), and San to French in Batondo, Kikigogo, and Sawa, respectively. The interpreter recorded the discussions while the GDs were taking place.

### Questionnaire interviews

As part of the ‘Behavioural and Environmental Assessment’ phase, surveys were conducted in 60 villages between February 2011 and January 2012. Since the main study is a community-based randomized control trial, the goal of the sampling was to compare the prevalence of active cysticercosis and the knowledge, attitudes and practices (KAP) relating to cysticercosis and taeniasis among participants through time. Therefore, the responses from the 80 participants per village are meant to represent KAP among those participants and are not meant to be generalized. However, we believe that the data represent the KAP pertaining to cysticercosis among people living in the 60 sampled villages and can be used to develop educational material for these communities. Such educational materials can be adapted to other endemic communities in the country.

**Fig. 1:**
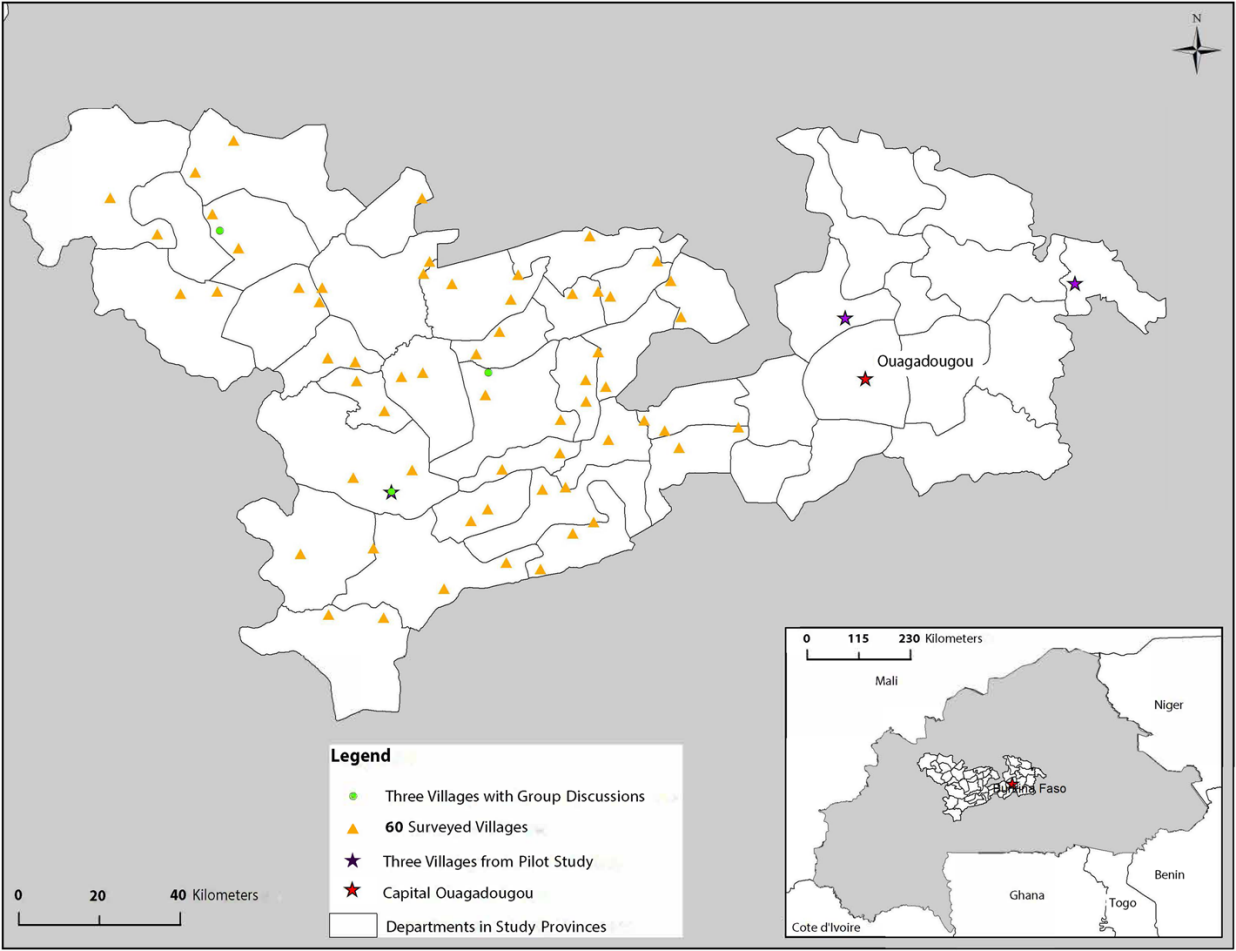
Villages used in the pilot study, GDs, and questionnaire survey during the planning of the health education intervention strategy to control taeniasis and cysticercosis in Burkina Faso, 2007–2012. One village (*green star*) was involved in both the pilot and the main study

Briefly, in each study province, all departments where pigs were raised (30 of 31 departments) were selected, and two villages meeting the eligibility criteria were randomly selected from each department for a total of 60 participating villages. Eligible villages had a population of at least 1 000 people as according to the 2006 census, were on the map provided by the Burkina Faso Geographic Institute (2000), were at least 5 km away from another participating village, were not located on a national or provincial road, were not the capital of the region or of the province, and were not located within 20 km of Koudougou or Ouagadougou. In each of the participating villages, 80 concessions were selected (a grouping of several households, usually members of the same family), including at least ten concessions with sows and at least 30 with pigs aged less than 12 months. One household was selected at random from each concession. In each sampled household, one eligible member was randomly selected to participate in a 3-year follow-up study on epilepsy and chronic headaches. As part of the follow-up study, participants had to answer a baseline questionnaire that was included in the present study (see Additional file 2). The inclusion criteria for participants in the baseline study and subsequent follow-up study were: aged at least 5 years, and having lived in the village for at least 12 months and not planning to move in the next 3 years. Figure 1 shows the location of villages used in the pilot study [4–6], the GDs, and the surveys.

The surveys were largely based on a cysticercosis questionnaire developed by the Cysticercosis Working Group in Eastern and Southern Africa (CWGESA). They measured sociodemographic factors, behaviors related to pork consumption, and knowledge on porcine cysticercosis, taeniasis, and of the life cycle of *T. solium*. In the first 40 selected concessions with sows or piglets, a blood sample was taken from one pig to measure the presence of active infection with cysticercosis. The owner of the sampled pig was asked to answer a questionnaire regarding pig management and knowledge on porcine cysticercosis. All data were recorded on personal digital assistants (HP iPAQ Rx5910 Travel Companion, USA). These electronic devices allowed better quality control during data collection and prevented potential errors during data entry.

### Data analysis

#### Development of thematic ideas and prevalence of various factors related to T. solium

The GD transcripts were team coded for recurring themes using NVivo 11.0 (QSR International, Americas) Inc. Burlington USA). Codes generally mirrored the content of the interview guide, permitting the retrieval of text on specific questions across interviews. The interview guide included a total of 36 questions. Thematic analysis identified nine primary codes, each with subthemes, accounting for a total of 51 identified subthemes.

The survey data were coded in Stata^®^ 13 (StataCorp LP College Station, Texas USA) and analysed in R version 3.1.3 [19] to estimate the frequency of the various factors assessed. Exact binomial 95% *CI*s were estimated for percentages with the binom command in R.

#### Rating behavioural and environmental factors related to T. solium

As part of the ‘Educational and Organizational Assessment’, the Health Belief Model (HBM) was used to rate the behavioural and environmental factors identified, focusing on their importance and potential for change through an education intervention. The HBM is an individual-level theory of health behaviour [20] developed to explain and predict health-related behaviours, particularly regarding the uptake of health services. Based on the GDs and surveys, predisposing, reinforcing, and enabling factors for prioritized behaviours, their importance, and potential for change were identified. Importance of a behaviour was based on how common a behaviour was found to be, as elucidated by the GDs and surveys. Changeability was assessed based on the GD participants’ perception of the possibility for behavioural change and some information from the survey. In addition, lessons learned from previous studies elsewhere guided the evaluation of any behaviour’s changeability. At this stage, the preliminary targets for the health education intervention were identified, focusing on predisposing, reinforcing, and enabling factors of at least moderate importance and changeability.

#### Identification of a suitable intervention approach

As part of the ‘Administrative and Policy Assessment’, available resources (financial, time, staff), policies, guidelines, and other political factors that could support or hinder the implementation of the planned interventions were assessed through a literature review and consultations with relevant organizations to identify suitable approaches for the current project. These included governmental organizations and non-governmental organizations (NGOs) that were involved in implementing community-based education programs in Burkina Faso.

## Results

### Perceptions related to T. solium based on the GDs

The results of the GD were used to complete the ‘Social Assessment’ phase of the PRECEDE model. The number and age range of the participants taking part in the 18 GDs are presented in Table 1. The number of participants was similar between the villages and each age category in each village also had similar numbers of participants. Batondo had previously participated in a pilot epidemiological study on human and porcine cysticercosis. However, based on the analysis of GD data from this study, familiarity with the *T. solium* life cycle in this village did not differ from that of Kikigogo and Sawa.

**Table 1:** Number and age range of participants taking part in the 18 GDs conducted in three villages of Burkina Faso, 2010

	*Village*							
	Batondo		Kikigogo		Sawa		Total	
*Participants*	No.	Age range	No.	Age range	No.	Age range	No.	Age range
Male youths	16	14–22	12	12–22	13	18–26	41	12–26
Female youths	11	14–20	16	14–20	16	17–26	43	14–26
Male adults	11	22–49	12	25–45	12	32–44	35	22–49
Female adults	13	25–47	13	24–46	16	30–45	42	24–47
Male older adults	8	50–70	12	45–70	13	45–76	33	45–76
Female older adults	11	44–60	18	45–85	12	45–61	41	44–85
Total	70	14–70	83	12–85	82	45–61	235	12–85

#### Perceptions about tapeworms in humans

Most people had seen worms in their faeces or those of others, but their description was insufficient to distinguish between worm species. The main source of human tapeworm infections was thought to be food. Some respondents mentioned inadequately cooked meat, some mentioned pork specifically: *“We think that it is badly cooked meat that we buy at the market which gives us the Necourdjomnè* [tapeworms]*”* (older female GD participant, Batondo); *“Any meat can give it, but especially pork meat, it is what we eat a lot of”* (adult male GD participant, Sawa). The respondents suggested several ways to avoid tapeworm infections, which included: avoiding the consumption of dirty food, improving personal and environmental hygiene, filtrating water, keeping pigs penned, and visiting healthcare centres for health check-up and deworming.

#### Pig rearing and pork consumption

Women were primarily responsible for raising pigs and managing income generated from pig sales, while men were more often involved in raising cattle, goats, and sheep. Women often received help from their children (especially daughters) who sometimes also owned pigs. The most common method of pig husbandry was to tie up pigs or pen them during the rainy season and let them roam during the dry season, with some variations noted. For example, some families let their pigs roam during the day and tied them up at night, others preferred to tie them up all the time, and some others would confine some pigs while letting others roam. Confinement during the rainy season was intended to prevent pigs from destroying crops. Penned pigs were typically fed grass, millet brew waste, and millet and maize bran. Roaming pigs were also given food but in smaller quantities than the confined pigs on anticipation that the roaming pigs source additional feed on their own when roaming. Most pigs drank from ponds but were given extra water from wells during the dry season. Respondents were aware that roaming pigs might eat human faeces and get illnesses, but most did not have pens or enough feed to prevent this from occurring. Participants recognized the positive value of keeping pigs penned to improve production, prevent diseases, avoid theft, and enhance collection of manure for land fertilization. Only two participants had received training about raising pigs. However, almost all of the participants, including men, were interested in such training to be provided during the dry season by nurses or through training someone from the village who would bring back the knowledge to the community. Participants were particularly interested in pig illnesses and their causes. Most of the participants said that they ate pork, which was generally prepared at home. Overall, pork was cheaper than lamb or beef, and easier to find in those localities. The most common and traditional way of preparing pork was to boil it in water with a few condiments, followed by a more recent technique of grilling it, cooking it in the oven, or frying it in oil.

#### Pig diseases

Participants reported two major diseases in pigs: one that caused white pimples to appear on the tongue and the rest of the body, though participants could not provide a name of the disease; and another that causes pigs to have seizures that suddenly start and manifested in shaking, drooling (and sometimes urinating), and falling. Many people referred to the latter illness as epilepsy. A few respondents explained that when realizing that a pig had epilepsy, they would try to sell it right away, hoping that the buyer would not detect its illness. Some mentioned that despite knowing the risks, many people ate the meat from epileptic pigs. Only two respondents from Sawa mentioned the risk of transmission, one saying that they would kill pigs with epilepsy to avoid transmission to other pigs. One respondent from Batondo explained that this illness had been on the rise over the past 2 years and that some people had lost all their pigs. Many people did not know the causes of pig epilepsy. A similar number of respondents offered explanations regarding causes of pig epilepsy, with the main one being food consumed, especially by roaming pigs, although no specific kind of food was mentioned. The unanimously identified solution to the problem was to build pens to prevent pigs from eating garbage and human faeces from the bush.

Another illness affecting pigs was the presence of white pimples. One common description was that pigs would stop eating, lose weight, and could die within a few days. Once their meat was cut, white pimples could be found on their bodies, under their tongues, and on their offal, such as their lungs and intestines. Respondents mentioned being told by the veterinarian to throw infected meat away and an abandoned well was identified as the prime location to dump carcasses. Respondents also mentioned that infected pigs were much more difficult to sell, the norm being to not want to purchase a pig with white dots under its tongue. The worst case scenario was to eat the infected meat since selling it at the market would be impossible. Participants were aware of the health-related risks of eating the meat, but they could not afford to throw it away. Some people identified filth and various other things that pigs find and eat in the bush as the cause of this disease, while others mentioned millet mixed with water or bran (the millet would accumulate in the pig’s body through time and would cause these pimples to appear). Aside from food, some participants believed that pigs were born with this disease, while other participants simply did not know the cause.

#### Open defecation

Due to a lack of latrines, it was found that open defecation is common in all villages, with both men and women defecating wherever they feel comfortable. The general trend is for people to defecate in the bush during the day, while staying closer to their house during the night. Other common defecation sites include under trees, abandoned houses, by a pond, or gardens, especially for families living far from the bush. It was found to be common for younger children to defecate behind the house or in a pot, the contents of which would later be discarded in the bushes or given to local dogs and pigs, whose role was to clean the areas surrounding the residences. Although open defecation was perceived as a shameful practice, it was also accepted as it has a long history that is difficult to change: *“It is embarrassing because a man can see you over there and he cannot greet you* [laughs]*”* (younger female GD participant, Kikigogo). People were very aware of the risk of open defecation for their health and that of their pigs. They were aware that having latrines was cleaner and reduced the spread of diseases by limiting the access of flies and animals to faeces. People said they would use latrines if they were available to them. Latrines were seldom available, except in schools (in Batondo) or ‘in town’, where people have to pay for them. Some proposed combining efforts as a solution. *“What I think is that if we want to build latrines, we can choose two or three households and build a latrine in the middle. Then we need to educate the members of the three households on how to use the latrines because some do not know that faeces must be put in the hole so they will do it on the side but if we show them, they will do it”* (older female GD participant, Kikigogo). Barriers to building latrines were mainly financial, with no cultural barriers found. Some respondents noted that a latrine constructed using only local materials would not be strong enough and could endanger its users. A good latrine in their opinion would be one made of cement, which was considered to be expensive.

#### Hand washing

Most people stated that they washed their hands only when they were about to eat, and some mentioned washing hands while washing their face in the morning or to get rid of dirt or right after defecation. A few people cited other timings: for prayer (Muslims), before cooking, during the sale of food, after shaking hands with someone, or after farm work. Most people mentioned washing their hands in order to avoid diseases and dirtiness. Many people could not afford soap and only used water to wash their hands.

#### Sources of drinking water

Most people obtained drinking water from open wells. The majority of these wells do not have an edge, which meant that the water is likely to be contaminated. Participants acknowledged that water from the tap or pump would have been the best but that only few people had access to this source. Villagers were very aware that drinking unclean water could cause stomachaches, malaria, epilepsy, guinea worm disease, intestinal worms, diarrhoea, and cholera. Additionally, participants, especially children, stated that using well water to wash themselves made their skin itch. The main solutions to having access to clean water that participants suggested was to build an edge with cement around the wells; though many said that they could not afford this.

In summary, the lack of latrines and safe water were considered potential problems compromising the quality of life of people in these villages. Human diseases such as epilepsy and poor access to healthcare services were also identified as problems. Pig production was an important enterprise that enabled women to meet family needs, but pigs scavenging on contaminated substances that contributed to zoonotic diseases were important constraints to pig production, limiting the pigs from expressing their full productivity potential because of poor nutrition. Lack of financial capacity was an important limitation to improved sanitation and pig management. Participants were very well aware of the health risks associated with poor sanitation, but were less knowledgeable about what caused pig diseases and their link to human health. Respondents were eager to learn about alternative methods of pig management and most claimed to have not had an opportunity for such training. There was a strong desire to build more latrines, though this was found to often be constrained by limited finances.

### Epidemiological assessment

The ‘Epidemiological Assessment’ phase of the PRECEDE model was completed during the pilot study outlined in the introduction [5, 6, 8–10] and demonstrated that cysticercosis was a health problem in Burkina Faso.

### Quantitative knowledge and practices related to T. solium based on questionnaire interviews

The results of the surveys were used to complete the ‘Behavioural and Environmental Assessment’ phase of the PRECEDE model. The sociodemographic characteristics of the 4 777 participants as well as of the 2 244 pig owners are summarized in Table 2. More than two-thirds of the participants never attended school and most were homemakers or farmers. The vast majority of pig owners were female.

**Table 2:** Sociodemographic characteristics of respondents answering the screening questionnaire assessing knowledge and practices related to *T. solium* and of interviewed pig owners in 60 villages in Burkina Faso, 2011–2012

Factor	Value	Percentage [95% *CI*]^a^
*Screening questionnaire*		
Province (*n* = 4 777)	Boulkiemdé	49.9 [48.5–51.4]
	Sanguié	33.3 [32.0–34.7]
	Nayala	16.7 [15.7–17.8]
Age distribution (*n =* 4 751)	≤15	28.0 [26.8–29.3]
	16–40	39.3 [37.9–40.7]
	≥41	32.7 [31.4–34.1]
Gender (*n* = 4 776)	Female	53.8 [52.4–55.2]
Education level (*n* = 4 774)	Attended school	29.0 [27.7–30.3]
School level (*n* = 4 775)	Incomplete primary school	87.5 [86.5–88.4]
	Completed primary school	7.8 [7.0–8.6]
	Completed secondary school	4.6 [4.0–5.2]
	Completed college	0.1 [0.06–0.3]
Occupation (*n* = 4 774)	Farmer	38.2 [36.8–39.6]
	Homemaker	35.1 [33.7–36.5]
	Student	19.4 [18.2–20.5]
	Other^b^	7.4 [6.7–8.2]
*Pig owner questionnaire*		
Province (*n* = 2 244)	Boulkiemdé	52.3 [50.2–54.4]
	Sanguié	35.0 [33.1–37.0]
	Nayala	12.7 [11.3–14.1]
Gender (*n* = 2 240)	Female	96.6 [95.8–97.3]

^a^binomial exact 95% *CI*

^b^e.g. commerce, salaried skills, unemployed

#### Participants’ knowledge on taeniasis and cysticercosis

Knowledge levels about how porcine cysticercosis or human taeniasis is acquired were quite low, and even lower among women (see Table 3). Among those who had heard or knew about human taeniasis, only 5.3% (95% *CI*: 4.5–6.1%) knew that the infection could be acquired by eating undercooked infected pig meat. Knowledge on how a human contracts tapeworm infection was significantly lower in Nayala than in Sanguié (prevalence proportion ratio, PPR: 0.2; 95% *CI*: 0.1–0.5). Knowledge on how a pig acquires cysticercosis was significantly lower in Boulkiemdé (PPR: 0.4; 95% *CI*: 0.3–0.5) and Nayala (PPR: 0.06; 95% *CI*: 0.02–0.20) compared to Sanguié. Males were more likely to possess knowledge on the route of tapeworm infection for humans and about how pigs acquire cysticercosis than females (PPR: 1.5; 95% *CI*: 1.1–2.0 and PPR: 2.2; 95% *CI*: 1.6–2.9, respectively) (see Table 3).

**Table 3:** Knowledge^a^ on taeniasis and porcine cysticercosis among questionnaire respondents from 60 villages in Burkina Faso, 2011–2012

Topic	Knowledge	Overall	Gender		Province		
			*Male*	*Female*	*Boulkiemdé*	*Sanguié*	*Nayala*
*Taeniasis*		*n* = 4 773	*n* = 2 205	*n* = 2 568	*n* = 2 385	*n* = 1 589	*n* = 799
Has heard of human tapeworm but has never been infected		53.4 [51.9–54.8]	53.1 [51.0–55.2]	53.6 [51.6–55.5]	50.8 [48.7–52.8]	57.5 [55.0–60.0]	52.8 [49.3–56.3]
Had a tapeworm infection/saw segments		9.4 [8.6–10.2]	10.7 [9.4–12.1]	8.2 [7.2–9.3]	12.5 [11.2–13.8]	3.7 [2.8–4.7]	11.5 [9.4–13.9]
Among those having heard of or having been infected with human tapeworm		*n* = 2 994	*n* = 1 407	*n* = 1 587	*n* = 1 508	*n* = 972	*n* = 514
Knowledge on how humans contract a tapeworm infection (eating undercooked pork)		5.3 [4.5–6.1]	6.4 [5.2–7.8]	4.3 [3.3–5.4]	5.4 [4.3–6.6]	7.1 [5.6–8.9]	1.6 [0.7–3.0]
Knowledge on how to recognize tapeworm infection (see worm in faeces)		49.2 [47.4–51.0]	50.0 [47.3–52.6]	48.6 [46.1–51.1]	43.4 [40.9–46.0]	57.3 [54.1–60.4]	51.0 [46.6–55.4]
Source of knowledge relating to human tapeworm (friend)		82.1 [80.7–83.5]	81.9 [79.8–83.9]	82.3 [80.3–84.1]	80.0 [77.9–82.0]	85.0 [82.6–87.2]	83.1 [79.5–86.2]
*Porcine cysticercosis*		*n* = 4 773	*n* = 2 205	*n* = 2 568	*n* = 2 385	*n* = 1 589	*n* = 799
Has heard about/seen pig cysts		63.4 [62.0–64.8]	64.7 [62.7–66.7]	62.3 [60.4–64.1]	69.9 [68.0–71.7]	58.8 [56.4–61.3]	53.2 [49.7–56.7]
Among those who have heard about/seen pig cysts		*n* = 3 026	*n* = 1 427	*n* = 1 599	*n* = 1 666	*n* = 935	*n* = 425
Knowledge on how a pig acquires cysts (eating human faeces)		6.2 [5.4–7.1]	8.7 [7.3–10.3]	4.0 [3.1–5.1]	4.4 [3.5–5.5]	12.0 [10.0–14.2]	0.7 [0.1–2.0]
Knowledge on where to find cysts in a live pig (under the tongue)		80.2 [78.7–81.6]	81.9 [79.8–83.9]	78.7 [76.6–80.7]	86.3 [84.5–87.9]	66.7 [63.6–69.8]	86.1 [82.5–89.3]
Source of knowledge on pig cysts (pig seller)		84.5 [83.1–85.7]	84.2 [82.2–86.1]	84.7 [82.8–86.4]	86.2 [84.4–87.8]	80.0 [77.3–82.5]	87.5 [84.0–90.5]

^a^Percentages and binomial exact 95% *CI*s

#### Knowledge and practices related to pig rearing and cysticercosis and taeniasis transmission

Most pig owners (89.5%) had heard about porcine cysticercosis (see Table 4). The percentage of pig owners reporting the ingestion of human faeces was the transmission route for porcine cysticercosis was much lower than among the questionnaire respondents. Pig owners used their profits from selling pigs to invest in business, pay for healthcare fees, and send children to school, rather than to buy food (see Table 4). Table 5 presents various practices related to *T. solium* transmission as reported by the questionnaire participants.

**Table 4:** Knowledge^a^ on porcine cysticercosis and profit objectives among current pig owners interviewed in 60 villages in Burkina Faso, 2011–2012

Topic	Knowledge	Overall	Gender		Province		
			*Male*	*Female*	*Boulkiemdé*	*Sanguié*	*Nayala*
*Porcine cysticercosis*		*n* = 2 242	*n* = 76	*n* = 2 162	*n* = 1 173	*n* = 785	*n* = 284
Has heard of porcine cysticercosis		89.5 [88.1–90.7]	92.1 [83.6–97.0]	89.4 [88.0–90.7]	87.6 [85.5–89.4]	90.4 [88.2–92.4]	94.7 [91.4–97.0]
Among those having heard of porcine cysticercosis		*n* = 2 006	*n* = 70	*n* = 1 933	*n* = 1 027	*n* = 710	*n* = 269
Knowledge on how a pig acquires porcine cysticercosis (eating human faeces)		0.1 [0.0–0.4]	0.0 [0.0–5.1]	0.1 [0.0–0.4]	0.0 [0–0.4]	0.3 [0.0–1.0]	0.0 [0.0–1.4]
Knowledge on where to find cysts in a live pig (under the tongue)		80.5 [78.7–82.2]	91.4 [82.3–96.8]	80.0 [78.2–81.2]	78.5 [75.8–81.0]	80.3 [77.2–83.1]	88.5 [84.0–92.0]
Among those who had knowledge on what to do with a pig with cysticercosis		*n* = 1 876	*n* = 58	*n* = 1 815	*n* = 939	*n* = 670	*n* = 267
What would you do if you were to discover nodules on your pig? (slaughter)		8.9 [7.7–10.3]	17.2 [8.6–29.4]	8.7 [7.4–10.0]	10.2 [8.4–12.3]	10.1 [8.0–12.7]	1.1 [0.2–3.2]
*Profits from pig farming*		*n* = 2 244	*n* = 76	*n* = 2 164	*n* = 1 174	*n* = 786	*n* = 284
To pay for healthcare fees		98.8 [98.2–99.2]	100.0 [95.3–100.0]	98.7 [98.1–99.1]	99.1 [98.4–99.6]	99.1 [98.2–99.6]	96.1 [93.2–98.1]
To invest in business		94.4 [93.4–95.3]	67.1 [55.4–77.5]	95.3 [94.4–96.2]	91.0 [89.2–92.5]	97.6 [96.3–98.5]	99.6 [98.1–100]
To send children to school		88.3 [86.9–89.6]	80.3 [69.5–88.5]	88.6 [87.2–89.9]	85.9 [83.7–87.8]	89.2 [86.8–91.3]	96.1 [93.2–98.1]
To buy food		0.7 [0.4–1.1]	5.3 [1.5–12.9]	0.5 [0.3–0.9]	1.3 [0.7–2.1]	0.0 [0.0–0.5]	0.0 [0.0–1.3]
*Pig housing management*		*n* = 2 243	*n* = 76	*n* = 2 163	*n* = 1 174	*n* = 785	*n* = 284
During the rainy season (penned)		44.8 [42.7–46.8]	47.4 [35.8–59.2]	44.6 [42.5–46.7]	44.1 [41.3–47.0]	41.1 [37.7–44.7]	57.4 [51.4–63.2]
During the dry season (roaming free)		98.7 [98.1–99.1]	85.5 [75.6–92.5]	99.2 [98.7–99.5]	98.4 [97.5–99.0]	99.0 [98.0–99.6]	99.3 [97.4–99.9]

^a^Percentages and binomial exact 95% *CI*s

**Table 5:** Prevalence (percentage) of practices related to taeniasis and porcine cysticercosis transmission in 60 villages in Burkina Faso, 2011–2012

Factor	Overall	Boulkiemdé province	Sanguié province	Nayala province
Primary source of drinking water (*n* = 4 754)				
Borehole (*n* = 2069)	43.5 (41.3–45.7)	58.0 (56.0–60.0)	30.6 (28.3–32.9)	25.8 (22.8–29.0)
Open well (1 930)	40.6 (38.4–42.9)	26.3 (24.5–28.1)	59.2% (56.8–61.6)	46.4% (42.9–50.0)
Drilled well or river water (755)	15.9 (13.4–18.7)	15.8 (14.4–17.3)	10.2 (8.7–11.8)	27.8 (24.7–31.9)
Boiling of drinking water (*n* = 4 774)				
Never (*n* = 4 738)	99.2 (99.0–99.5)	98.8 (98.2–99.2)	99.6 (99.2–99.9)	100 (99.5–100)
Sometimes to always (*n* = 36)	0.8 (0.5–1.0)	1.2 (0.8–1.7)	0.4 (0.1–0.8)	0 (0–0.5)
Eat pork (*n* = 4 772)				
Yes (*n* = 3 156)	66.1 (64.8–67.5)	66.6% (64.6–68.5)	72.5% (70.2–74.6)	52.3% (48.8–55.8)
Boiling pork is the preferred style (*n* = 3 156)	98.3 (97.7–98.7)	97.7% (96.9–98.4)	98.6% (97.8–99.2)	99.3% (97.9–99.9)
Eat pork outside their homes at least some of the time (*n* = 3 156)	43.4 (42.1–44.8)	43.9% (41.4–46.4)	59.7% (56.7–62.6)	40.7% (35.9–45.6)
Do not use latrines to defecate (*n* = 4 774)		86.5% (85.1–87.8)	90.6% (89.1–92.0)	85.7% (83.1–88.1)

### Evaluating the potential for behavioural change and changing environmental factors

The results from the ‘Social Assessment’, the questionnaire interviews, and findings from similar studies conducted elsewhere [21–23] were used to rate the importance and evaluate the potential for change in behaviour and the environmental factors identified (see Table 6). This evaluation is part of the ‘Behavioural and Environmental Assessment’.

**Table 6:** Behavioural and environmental factors related to the prevalence of human and porcine cysticercosis, their importance, and potential for change, Burkina Faso, 2007–2012

Determinant*	Type	Importance	Changeability
Open defecation	Behavioural	High	Low
Drinking unboiled water	Behavioural	High	Low
Consumption of undercooked pork*	Behavioural	High	Moderate
Lack of latrines*	Environmental	High	Moderate
Allowing pigs to roam	Behavioural	High	Low
Seasonality of pig feeds	Environmental	High	Low

*Prioritized

Open defecation was a highly prevalent behaviour reported during the GDs and questionnaire interviews, and is strongly associated with the prevalence of human cysticercosis epidemiologically [21]. While an education program strictly focused on reducing open defecation was expected to only slightly reduce this behaviour due to a lack of latrines, the lack of perceived cultural barriers to constructing latrines suggests that including training on how to build latrines into the education program could improve the situation. In addition, it is possible that being aware that epilepsy can be prevented through better sanitation could convince people to invest in building latrines. Hence, latrine ownership was assessed as being potentially changeable (Table 6) if the community was provided with the skills on how to construct durable latrines using locally available, less expensive materials and if given more knowledge on the link between sanitation and neurocysticercosis-associated epilepsy. Adoption of latrine use has been realized in some parts of the country where the government and some NGOs have assisted in the building of latrines for communities through local projects.

Eating undercooked pork was considered moderately changeable if education on the health risks associated with this behaviour was provided. Indeed, consuming pork at village markets was identified as being strongly associated with human cysticercosis [21], even though the reporting of eating undercooked pork was not frequent. It is believed that pork prepared at village markets is more likely to be undercooked because this is most practiced in areas where most of the consumers are drinking alcohol, commonly at peripheries of the market place. In this case it is possible that villagers may not report the level of pork cooking accurately. Hence, the importance of thoroughly cooking pork was prioritized for the education program.

In addition to being weakly associated with the prevalence of human cysticercosis infection [21], allowing pigs to roam was considered less amenable to change given the requirement for financial investment in fencing, which was perceived as being difficult in this financially constrained community. In addition, the potential to change the problem of feed seasonality was rated as low due to the financial and work burdens this would place on participants in feeding the confined pigs indoors.

Drinking unboiled water was considered to have low changeability given its requirement for financial investment such as cost of fuel.

### Predisposing, reinforcing, and enabling factors for lack of latrines and consumption of undercooked pork

The results of the ‘Social’ and ‘Behavioural and Environmental’ assessments were used to identify and rate the predisposing, reinforcing, and enabling factors for behaviours deemed changeable (i.e. the building of latrines and pork consumption) for their importance and changeability during the ‘Educational and Organizational Assessment’ phase. Three predisposing and two reinforcing factors, and one enabling factor were identified (see Table 7).

**Table 7:** Predisposing, reinforcing, and enabling factors related to open defecation, drinking unboiled water, and consumption of infected pork, Burkina Faso, 2007–2012

Determinant*	Type	Importance	Changeability
Knowledge on T. solium*	Predisposing	High	High
Perceived financial benefits of porcine cysticercosis control*	Predisposing	High	High
Public sensitization*	Reinforcing	High	Moderate
Perceived financial barriers	Predisposing	High	Low
Selfefficacy*	Reinforcing	High	Unknown
Health extension services	Enabling	High	Unknown

*Prioritized

The first predisposing factor was a lack of knowledge on the life cycle and impact of *T. solium*. Indeed, people did not have a great deal of knowledge on their susceptibility or the severity of infections that could be acquired from pigs. The knowledge gap was considered highly changeable, as it could be easily addressed through education [22–24]. The link between *T. solium* and epilepsy in humans should be emphasized, as the community already has strong fears about the impacts of epilepsy.

A second predisposing factor was the perceived financial benefits of pigs and the profits from their sale being used to pay for healthcare fees, invest in business, and send children to school. These benefits could be emphasized to motivate control efforts.

The third predisposing factor was the perceived financial burden of implementing *T. solium* control measures such as building latrines or confining pigs. This could prove to be a major barrier, resulting in this factor being deemed low in terms of changeability.

Self-efficacy was considered a potential re-enforcing factor for preventing *T. solium* transmission that was lacking in this resource-poor community, as revealed by the community’s strong belief that they would not be able to construct durable latrines or pigpens using local materials. A community-led total sanitation model (CLTS) [25], although it does not prescribe latrine styles, is perceived to be an important model to enhance self-efficacy, as participants can learn from each other when they decide to build latrines to stop open defecation. However, the rate of change of self-efficacy was unknown as the CLTS model itself had not yet been evaluated under controlled trials. Nevertheless, self-efficacy was prioritized in this study.

Another important reinforcing factor lacking in the community was public sensitization. Apart from a few mentions of nurses as sources of health messages, there was no other source of health communication mentioned during the GDs. Similarly, the questionnaire indicated that the major source of information on taeniasis and porcine cysticercosis were citizens rather than health professionals or schools.

Health extension services were considered as an important enabling factor for initiating and sustaining behavioural change that was lacking in this community. It was apparent that neither health nor veterinary extension agents provided education to the villagers regarding taeniasis or cysticercosis, as more than 80% of respondents reported to have acquired their knowledge from friends or pig sellers (see Table 3).

Public sensitization could be improved through the community-based model as well as through the production of a comedy film and comic book about safe pig management, the importance of latrine use, and epilepsy. The comedy films and comic books are particularly effective as many people enjoy following the stories given in these materials if they are attractive. In addition, because such kind of media show actions more than words, they are understood by many people, including illiterates.

### Intervention priorities

The last phase of the PRECEDE approach is to conduct an ‘Administrative and Policy Assessment’ to identify and adjust administrative and policy issues that could affect the successful implementation of the planned interventions. In addition, the resources available (including time and budget) should be assessed in relation to the planned intervention activities.

The planned sanitation-based interventions in this study were well matched to the water and sanitation policies of Burkina Faso. Unfortunately, the implementation of the initially planned community model, the CLTS, faced several hurdles. Upon consultation with Water and Sanitation for Africa, an NGO in Burkina Faso experienced in CLTS implementation, it was found that the CLTS implementation would be prohibitively expensive to implement and not sustainable. A less expensive and resource-intensive community model referred to as Participatory Hygiene and Sanitation Transformation (PHAST) was proposed. The PHAST methodology [26] aims to improve hygiene-related behaviours of communities and stimulate their management of water and sanitation facilities as well as to prevent diarrheal diseases. The PHAST initiative uses Self-esteem, Associative strengths, Resourcefulness, Action planning, and Responsibility (SARAR) as its underlying participatory methodology. A basic principle of SARAR is the recognition and affirmation of people’s innate abilities [26]. Two main principles are: (i) people will solve their own problems best in a participatory group process and (ii) the group collectively will have enough information and experience to begin to address its own problems. SARAR also recognizes that self-esteem is a prerequisite to decision-making and performance. The PHAST concept had been extensively tested, improved, and subsequently applied in several countries including Zimbabwe [27], Kenya [28, 29], Uganda [30], and Uzbekistan [31]. Therefore, the project adopted the PHAST-SARAR as the community mobilization model instead of CLTS.

In summary, four factors were prioritized for the intervention, namely (1) knowledge on *T. solium* transmission, impact and control; (2) perceived financial benefits of controlling porcine cysticercosis; (3) public sensitization; and (4) self-efficacy, particularly regarding the construction of latrines.

#### Development of a comedy film and accompanying comic book

It was also found vital to combine the PHAST method with the production of a comedy film and an accompanying comic book based on the film and written by the screenwriter. These material would increase knowledge to complement PHAST which is aimed primarily at enhancement of action competence. The 52-min film and the comic book are aimed at informing audiences about the importance of latrine use, education about epilepsy, and safe pig management, to enhance adoption of *T. solium* control measures, especially the use of latrines. The prioritizing factors and basic information on the life cycle of *T. solium* were shared with the screenwriter.

The film features several main characters: a pig trader, a nurse, a veterinarian, a pig farmer, a person living with epilepsy, the son of an imam, male village elders, village women, and extras. The extras are used to set the scene about how people traditionally believe epilepsy is caused. The male elders, son of the imam, nurse, veterinarian, and the village women are used to educate the audience about the importance of building latrines to prevent diseases, restrict pig access to human faeces, and thus indirectly prevent epilepsy, as well as the importance of cleaning food before eating it and of hand washing. The pig trader character is used to educate the audience about the dangers of selling infected pigs, letting pigs roam free, and of eating undercooked meat. The pig farmer character is used to demonstrate improved pig management techniques and their benefits. The person with epilepsy, nurse, and veterinarian are used to reduce the stigma associated with epilepsy and to educate people about the link between poor sanitation and epilepsy.

The film shows how all members of a village decide to take their health into their own hands by building latrines after being pressured to do so by women who have been educated by the veterinarian and nurse about the threats of open defecation. The women pressure the men by going on a “bed strike” until the latrines are built. The pig trader is at first presented as “cheating” the meat inspection system and arguing against control epilepsy to becoming a driver for latrine building and proper pork cooking. Each step of the evolution of this character is presented humorously. The son of the imam is also a funny character who drinks alcohol and eats pork, sometimes undercooked. He acquires taeniasis due to his pork consumption habits and defecates in the bush without washing his hands. The pig farmer, because of his relative prosperity from pig raising and not having anyone with epilepsy in his family, is accused by some of the village elders of having put a spell on the epilepsy patient, an accusation which is demystified through education, with the pig farmer teaching one of the elders how to build solid pigpens. These are just a few examples of the different plots in the film to disseminate the messages.

**Fig. 2:**
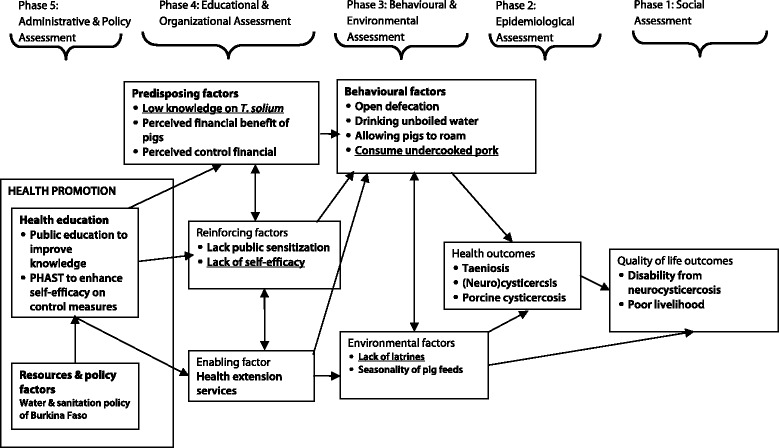
PRECEDE model for controlling *T. solium* taeniasis and cysticercosis in Burkina Faso planned using an implementation research method, 2007–2012. The PRECEDE phases can be read from right to left. **Phases 1 and 2**: The quality of life of the study community is compromised by the prevalence of human and porcine cysticercosis. The community desires access to safe water, latrines, and healthcare services, all of which can be linked to diseases. An intervention to reduce prevalence of not only cysticercosis but other diseases is linked to poor sanitation, and safe water is desired. **Phase 3**: Significant and sustainable improvements in the behavioural and environmental factors are necessary to reduce the frequency of human and porcine cysticercosis. Construction and use of latrines needs to be promoted to stop open defecation. Prevention of consumption of undercooked pork could be managed by an education intervention. **Phase 4**: Cysticercosis is predisposed by a lack of knowledge on *T. solium* and the advantages of its control. Lack of self-efficacy can be the reason why households are not constructing latrines or pigpens. Health extension services are important in the initiation and maintenance of behavioural and environmental factor changes. **Phase 5**: A health promotion program in the study area should focus on improving knowledge and enhancing self-efficacy in implementing *T. solium* control measures. The developed intervention strategy consists of a 52-min film and accompanying comic booklet to improve knowledge and PHAST to enhance self-efficacy. Water and sanitation policy for the Burkina Faso government and that of NGOs are consistent with the initiative of this study. For example, several organizations, including UNICEF, WaterAid, Plan International and World Bank have been supporting initiatives focusing on improving community access to safe water, sanitation and hygiene in Burkina Faso for many years. The health education strategy is expected to improve knowledge on *T. solium* and self-efficacy in the construction of pit latrines. These in turn will reduce risk behaviours related to *T. solium* transmission. Consequently, the prevalence of taeniasis and cysticercosis will be reduced. This will contribute to the improvement of quality of life of the community

Most actors are very well known television stars in Burkina Faso and both the screenwriter (Noraogo Sawadogo) and film director (Missa Hébié) have won several international awards for their work. Both the film and comic book were made more pictorial than the textual to enable illiterate audiences to grasp the key messages. The intervention would target the general population taking into account the zoonotic implication of *T. solium*. The aims of the intervention will be to (i) improve community knowledge on *T. solium* transmission, impacts, and benefits of control measures and (ii) improve self-efficacy (through the PHAST method) in implementing control measures such as building durable latrines and pig houses using local resources (see Fig. 2).

## Discussion

This study used an implementation research method to plan the development of an education intervention for the control of cysticercosis and taeniasis in Burkina Faso. The use of theory-based interventions, evaluated through appropriate designs, contributes to the understanding of why interventions “work” or not under particular conditions [32]. It calls for the need to utilize the explanatory and predictive capability of theory to the design of both programmes and evaluations. Research has shown that health education interventions that are based on sound theoretical frameworks are most likely to be effective [12].

The PRECEDE model has had many applications in research and development activities. In the USA, an infection prevention promotion program based on the PRECEDE model significantly and sustainably improved hand hygiene behaviours among healthcare personnel [33]. Two Australian case studies found that PRECEDE was a strong theoretical model that guided the development of realistic nurse-led interventions with the best chance of being successful in existing healthcare environments [34]. A study in Nepal used the PRECEDE model to define actions necessary to control *T. solium* in the country. The authors identified hurdles in administration and policy, where standardized law-enforcement and meat inspection practices were needed [35]. A similar strategy in Tanzania reduced the incidence rate of porcine cysticercosis by 43% and significantly reduced reported consumption of infected pork. However, the intervention did not increase the use of latrines or pig confinement [16, 22]. This intervention focused on pig management and was based only on health communication, which most probably was not enough to motivate and improve self-efficacy in the implementation of disease control actions, especially the use of latrines.

In the current study, a comprehensive assessment of the study community led to a considerable understanding of the needs, strengths, and weaknesses in the control of *T. solium* infections in Burkina Faso. Taking into account lessons learned in similar studies conducted elsewhere, the authors of this study were able to plan an evidence-based health education strategy for controlling *T. solium* in three provinces. Incorporation of the PHAST strategy was presumed to improve the adoption of latrine use, encourage the building of latrines, and consequently reduce open defecation. Previous experience of disseminating research findings using forum theatre’s in the country had already shown the capacity of communities to capture key messages. In the present study, instead of forum theatre’s, which would require to be played several times, and hence would be more costly, a 52-min film produced locally was adopted. The advantage is that the film could be played as many times as desired without variations in content and quality, and would not require extensive human resource for its dissemination. It was expected that the PHAST strategy combined with the film would provide communities with the information necessary to control *T. solium*.

One of the limitations of this study is that some useful information might have been missed in the GDs because of the note-based method used to record the responses. In addition, the direct translation and back translation of the questions at the time of data collection might have led to some information being lost. Furthermore, the questionnaire used was not directly validated. However, the study employed an extensively used questionnaire and validity was assessed through triangulation with GDs, which gives the authors confidence that the questionnaire captured the KAP appropriately.

## Conclusions

Both qualitative and quantitative methods pointed to a lack of knowledge on taeniasis and cysticercosis, as well as ongoing problems maintaining hygiene and sanitation sufficient to avoid human and porcine cysticercosis, in villages in the three studied provinces of Burkina Faso. Reasons for the observed significant differences between provinces related to some of the knowledge could not be established in this study, though it does emphasize the need to highlight different aspects when implementing health education interventions in these provinces. What these communities had in common was reliance on pork as a food source from pigs that were largely free-roaming and scavenging on refuse in the local environment, including on human faeces that might be contaminated with *T. solium* eggs. Relying on a free-roaming animal that is able to obtain nourishment from whatever food sources are easily available rather than feeding it purchased animal feed (and which also performs the ecoservice of cleaning the local environment) is an economically rational strategy for an impoverished population, and one which, as the PRECEDE model determined, was unlikely to be easily changed. We believe that the larger issue in these communities with regard to the transmission of *T. solium* cysticercosis is the availability of contaminated faeces for consumption by these animals, the prevention of which requires the construction of latrines, which is quite problematic in an economically challenged setting. Providing the community with the skills to construct durable latrines using low-cost locally available materials would likely help to resolve this problem. If the environment is free from human faeces, most of the other behaviours (such as drinking unboiled water, letting pigs roam free, and eating partially cooked pork) would have a low impact in the transmission of the parasite. Further studies are required to implement and evaluate the *T. solium* control strategy developed in this study.

## Additional files

Additional file 1: Multilingual abstract in the five official working languages of the United Nations. (PDF 641 kb)

Additional file 2: A questionnaire to assess participants’ knowledge and practices related to Taenia solium cysticercosis and taeniasis in 60 villages in Burkina Faso between Feburary 2011 and January 2012. (DOC 49 kb)

## Abbreviations

Ag-ELISA: Antigen enzyme-linked immunosorbent assay
BCI: Bayesian credible interval
CI: Confidence interval
CLTS: Community-led total sanitation
GD: Group discussion
HBM: Health Belief Model
NGO: Non-governmental organization
PHAST: Participatory Hygiene and Sanitation Transformation
PPR: Prevalence proportion ratio
PRECEDE: Predisposing, Reinforcing, and Enabling Constructs in Educational Diagnosis and Evaluation
SARAR: Self-esteem, Associative strengths, Resourcefulness, Action planning, and Responsibility
