# Supplementary material for: Development of a health education intervention strategy using an implementation research method to control taeniasis and cysticercosis in Burkina Faso
Source: Infect Dis Poverty. 2017 Jun 1;6:95. doi: 10.1186/s40249-017-0308-0 (PMC5452375; doi:10.1186/s40249-017-0308-0)
Supplement: Supplementary file 1 — Multilingual abstract in the five official working languages of the United Nations. (PDF 641 kb) [file 40249_2017_308_MOESM1_ESM.pdf]

Translation of the abstract into the five official working languages of the United Nations

بوركينا فاسووضع استراتيجية تثقيف صحي للتدخل باستخدام طريقة بحث تنفيذية للسيطرة على داء الشريطيات وداء الكيسات المذنبة في

هيلينا نجووي، إيفان أوزبولت، أثاناس ميلوجو، فيرونيك ديرموو، تيليسفور سومي، بول سبيسر، لوري ل. جيرفيس، راسماني جانابا، سارة جبرائيل، بيير دورني، هيلين كارابين

ملخص

خلفية: الشريطيات وداء الكيسات المذنبة هما الأمراض الناجمة عن الشريطية الوحيدة، وهي طفيلي ينتقل بين البشر والخنازير، مما يؤدي إلى خسائر اقتصادية كبيرة ولحدوث إعاقات. ويرتبط انتقال الطفيل إلى العوامل البيئية والسلوكية مثل عدم كفاية المرافق الصحية والنظافة، وسوء التعامل مع الخنازير، واستهلاك لحم الخنازير المصابة. هذه الدراسة استخدمت طريقة البحث التنفيذي لتصميم استراتيجية تثقيف صحي للتدخل للحد من التهاباتالشريطية الوحيد في بوركينا فاسو، البلد التي يتوطن فيها هذا الطفيلي.

الطرق: أجريت ثمانية عشر مناقشات جماعية مع 8-18 مشارك في ثلاث قرى. وبالإضافة إلى ذلك، تم إجراء مقابلات منظمة بين 4777 مشاركا و 2244 من أصحاب الخنازير، الذين تم اختيارهم من خلال العينة العشوائية العنقودية في 60 قرية من ثلاث محافظات في بوركينا فاسو. كلا النهجين قيم المعارف والممارسات المتصلة بطفيل الشريطية الوحيدة. تم استخدام المعلومات التي تم الحصول عليها لوضع استراتيجية تثقيف صحي للتدخل تتكيف مع المجتمع للسيطرة على داء الشريطيات وداء الكيسات المذنبة في بوركينا فاسو.

النتائج: كشفت مناقشات المجموعة أن المشاركين يعيشون في مستوى حياة متدن بسبب الأمراض وكذلك صعوبة الوصول إلى المراحيض، والمياه الصالحة للشرب، وخدمات الرعاية الصحية. وبالإضافة إلى ذلك، فقد وجد أن إنتاج الخنازير كان نشاطا اقتصاديا هاما، خاصة بالنسبة للنساء. وعلاوة على ذلك، كانت القيود المالية ومعرفة أوجه القصور الهامة لتحسين التعامل مع الخنازير وبناء المراحيض. وأظهرت بيانات المسح أيضا أن التغوط في العراء وشرب الماء غير المغلي كانت السلوكيات المشتركة، وعززت من نقص المعرفة فيما يتعلق بنقل الطفيل، وعجز التمويل لتنفيذ تدابير الرقابة، وعدم التوعية العامة، فضلا عن عدم وجود الكفاءة الذاتية نحو السيطرة على الطفيليات. ومع ذلك، يمكن التأكيد على المنافع التي ستتأتى من توفير التمويل للسيطرة على داء الكيسات المذنبة المنقول من خلال الخنازير عن طريق برنامج تعليمي يشجع على تجنب التغوط في العراء وتشجيع شرب المياه الصالحة للشرب. وتضمنت استراتيجية التدخل النهائية منهجية المشاركة في تحسين الممارسات المتعلقة بالنظافة الصحية ومرافق الصرف (PHAST)، وكذلك فيلم مدته 52 دقيقة وكتيب مصور مرفق به.

الاستنتاجات: المشكلة الرئيسية في مجتمعات الدراسة فيما يتعلق بنقل داء الكيسات المذنبة الذي تسببه الشريطية الوحيدة هو التخلص العشوائي من براز الإنسان، والتي يمكن أن يكون ملوثا ببيض الطفيلي. منع التغوط في العراء يتطلب بناء المراحيض، والتي يمكن أن تكون مشكلة كبيرة من الناحية الاقتصادية. تزويد المجتمع بالمهارات اللازمة لبناء مراحيض دائمة باستخدام المواد منخفضة التكلفة المتاحة محليا من شأنه أن يساعد على الأرجح في حل هذه المشكلة. هناك حاجة لدراسات إضافية لتنفيذ وقييم استراتيجية مكافحة الشريطية الوحيدة المعدة في هذه الدراسة.

Translated from English version into Arabic by datran, through

TRANSLATORS
WITHOUT BORDERS

布基纳法索绦虫病和囊尾蚴病防控中卫生教育干预策略的制定

Helena Ngowi, Ivan Ozbolt, Athanase Millogo, Veronique Dermauw, Télesphore Somé, Paul Spicer, Lori L. Jervis, Rasmané Ganaba, Sarah Gabriel, Pierre Dorny, Hélène Carabin

摘要
引言：绦虫病和囊尾蚴病均由猪带绦虫（*Taenia solium*）引起的，该虫在人与猪之间传播，给养猪业造成巨大的经济损失，也给人类身体健康带来危害。猪带绦虫的传播与环境和行为因素相关，如卫生设施和条件不足，养猪管理不善，感染猪肉流入市场等。本研究针对该寄生虫感染高发国布基纳法索制定了卫生教育干预策略，并采用实施法进行研究，以降低猪带绦虫在该地区的感染率。
方法：在 3 个村庄选取 18 组进行调查，每组 8-18 名参与者。此外在布基纳法索 3 个省的 60 个村庄，随机抽样选取 4 777 名参与者和 2 244 位养猪户进行结构性访谈。这两种方法旨在评估居民对猪带绦虫的认识和预防措施。获取的信息用于制定适用于社区的卫生教育干预策略，以控制布基纳法索当地的绦虫病和囊尾蚴病。
结果：小组调查显示，由于该两种寄生虫病的困扰，加之缺乏安全的饮用水、厕所和卫生保健服务，参与者的生活质量受到严重影响。调查还发现养猪业在当地，尤其对妇女而言，是重要的经济活动。此外，财政困难和知识匮乏是改善养猪管理和推进公厕建设的重要限制性因素。调查数据还表明，由于缺乏寄生虫传播有关知识，现有经济门槛阻碍防控措施落实，公共宣传力度不足，以及缺乏对寄生虫控制的自我效能感，使得随地排泄、饮用生水等行为在当地十分普遍。尽管如此，仍可以通过教育引导人们杜绝随地排泄，饮用清洁水，进一步强调防控猪囊尾蚴病所带来的经济收益。最终的干预策略包括参与性清洁卫生改造（PHAST），以及一部 52 min 的短片和相应的漫画宣传册。
结论：本研究涉及到的社区中，猪囊尾蚴病传播相关的主要问题是人类粪便的不当处理，包括寄生虫感染者带有虫卵的粪便。杜绝随地排泄行为依靠厕所建设，但对经济拮据的贫困地区而言困难重重。而向社区提供技术，使用本地低成本材料建设坚固耐用的厕所，可能更有利于解决该问题。有关本研究制定的猪带绦虫的控制策略的落实和评价，仍需进一步研究。

Translated from English version into Chinese by Liang Jia-Qi, edited by Yang Pin

Développement d'une stratégie d'intervention d'éducation en santé utilisant la mise en œuvre d'une méthode de recherche pour contrôler la téniasis et la cysticercose au Burkina Faso

Helena Ngowi, Ivan Ozbolt, Athanase Millogo, Veronique Dermauw, Télesphore Somé, Paul Spicer, Lori L. Jervis, Rasmané Ganaba, Sarah Gabriel, Pierre Dorny, Hélène Carabin

Résumé
Historique : La téniase et la cysticercose sont deux maladies causées par le *Taenia solium*, un parasite transmis entre les humains et les porcs, conduisant à des pertes économiques considérables et des infirmités. La transmission du parasite est liée à des facteurs environnementaux et comportementaux, tels qu'une désinfection et une hygiène inadéquate, une mauvaise gestion des porcs, ainsi que la consommation de viande de porc infectée. Cette étude a eu recours à la mise en œuvre d'une méthode de recherche visant à concevoir une stratégie d'intervention d'éducation en santé pour réduire les infections du *T. solium* au Burkina Faso, un pays endémique pour le parasite.
Méthodes : Dix-huit discussions de groupe ont été menées auprès de 8 à 18 participants dans chacun des trois villages. De surcroît, des entrevues structurées ont été menées parmi 4777 participants et 2244 propriétaires de porcs, qui ont été sélectionnés par groupe d'échantillonnage aléatoire dans 60

villages de trois provinces du Burkina Faso. Les deux approches ont évalué les connaissances et pratiques liées au *T. solium*. L'information obtenue a servi à élaborer une stratégie d'intervention en éducation de la santé adaptée à la communauté afin de contrôler la téniase et la cysticercose au Burkina Faso.

Résultats : les groupes de discussion ont révélé que les participants avaient une mauvaise qualité de vie en raison de maladies, ainsi qu'un accès inadéquat à des latrines, de l'eau salubre et des services de soins de santé. En outre, il a été constaté que la production porcine était une activité économique importante, particulièrement pour les femmes. De plus, les contraintes et les connaissances financières constituaient une importante limitation à l'amélioration de la gestion des porcs et de la construction de latrines. Les données du sondage ont également indiqué que défécation en plein air, ainsi que la consommation d'eau potable non bouillie, étaient des comportements communs, renforcés par un manque de connaissances en ce qui concerne la transmission de parasite, perçu les obstacles financiers à la mise en œuvre des mesures de contrôle, le manque de sensibilisation du public, ainsi que le manque de connaissances en ses propres capacités quant au contrôle du parasite. Néanmoins, les avantages financiers perçus du contrôle de la cysticercose porcine pourraient être améliorés par un programme d'éducation décourageant la défécation en plein air et invitant à consommer une eau salubre. La stratégie d'intervention finale incluait une approche à l'hygiène participative et l'assainissement (PHAST), ainsi qu'un film de 52 minutes et un livret de bande dessinée d'accompagnement.

Conclusions : le principal problème dans les communautés étudiées quant à la transmission du *T. solium* cysticercose est la disposition aléatoire des matières fécales humaines pouvant être contaminées par des œufs de parasite. La prévention de la défécation en plein air nécessite la construction de latrines, ce qui peut être passablement problématique dans les milieux économiquement défavorisés. Offrir à la communauté les compétences nécessaires pour construire des latrines durables avec des matériaux locaux de faible coût serait susceptible de les aider à résoudre ce problème. D'autres études sont nécessaires à mettre en œuvre et à évaluer la stratégie de contrôle du *T. solium* développé dans cette étude.

Translated from English version into French by lisaguay, through

TRANSLATORS
WITHOUT BORDERS

Разработка стратегии внедрения санитарного просвещения для контроля над распространением тениоза и цистицеркоза в Буркина-Фасо посредством исследовательского подхода

Хелена Нгови, Иван Озболт, Атанасэ Милого, Вероник Дермоу, Телесфора Соме, Пол Спайсер, Лори Л. Джервис, Расмане Ганаба, Сара Габриэль, Пьер Дорни, Элен Карабин

Аннотация

Справочная информация: Тениоз и цистицеркоз представляют собой заболевания,

вызываемые свиным солитером (*Taenia solium*). Данный паразит передается между людьми и свиньями и приводит к инвалидности и значительным экономическим потерям. Передача паразита связана с экологическими и поведенческими факторами, например, низким уровнем санитарии и гигиены, ненадлежащим управлением в свиноводстве и потреблением зараженной свинины. Данная работа использует исследовательский подход для разработки стратегии внедрения санитарного просвещения для снижения заражения паразитом *T. Solium* в Буркина-Фасо, государстве, эндемичном для паразита.

Методы: в каждой из трех деревень были проведены восемнадцать групповых обсуждений, включающих от 8 до 18 участников. Кроме того, были проведены структурированные интервью 4 777 участников и 2 244 свиновладельцев, отобранных методом случайной кластерной выборки в 60 деревнях трех провинций Буркина-Фасо. Оба подхода использовались для оценки знаний и практик, связанных с *T. Solium*. Полученная информация использовалась для разработки стратегии внедрения адаптированного к местным реалиям санитарного просвещения для контроля над распространением тениоза и цистицеркоза в Буркина-Фасо.

Результаты: групповые обсуждения показали низкое качество жизни участников вследствие болезней, а также недостаточного доступа к туалетам, безопасной воде и медицинским услугам. Кроме того, было установлено, что производство свинины является важной экономической деятельностью, особенно для женщин. Более того, ограниченные финансовые возможности и знания являются серьезными преградами на пути улучшения процессов свиноводства и сооружения туалетов. Данные исследования также показали, что открытая дефекация и питье сырой воды являются типичным поведением, усугубляемым отсутствием знаний о способах передачи паразита, существующими финансовыми ограничениями для осуществления мер контроля, отсутствием широкой разъяснительной работы, а также отсутствием самоэффективности направленной на контроль над распространением паразита. Тем не менее, выявленные финансовые преимущества контроля над цистицеркозом свиней могут быть усилены образовательной программой, которая дестимулирует открытую дефекацию и поощряет употребление кипяченой воды. Итоговая стратегия вмешательства включала совместное изменение санитарных и гигиенических условий (PHAST-подход), а также создание 52-минутного фильма и сопровождающего его иллюстрированного буклета.

Выводы: основную проблему, связанную с передачей вызывающего цистицеркоз паразита *T. solium*, в исследуемых сообществах составляет произвольное удаление человеческих фекалий, которые могут быть заражены яйцами паразита. Предотвращение открытой дефекации требует сооружения туалетов, что может быть весьма проблематичным в экономически сложных условиях. Обучение сообщества навыкам строительства прочный туалетов, с использованием недорогих местных материалов, скорее всего, поможет решить данную проблему. Необходимы дальнейшие исследования для внедрения предложенной в настоящей работе стратегии контроля над *T. Solium* и оценки ее эффективности.

Translated from English version into Russian by datran, through

TRANSLATORS WITHOUT BORDERS

Desarrollo de una estrategia de intervención de educación sanitaria utilizando un método de aplicación de investigación para el control de la teniasis y la cisticercosis en Burkina Faso

Helena Ngowi, Ivan Ozbolt, Athanase Millogo, Veronique Dermauw, Télesphore Somé, Paul Spicer, Lori L. Jervis, Rasmané Ganaba, Sarah Gabriel, Pierre Dorny, Hélène Carabin

Resumen
Información de referencia: La teniasis y la cisticercosis son dos enfermedades causadas por Taenia solium, un parásito transmitido entre humanos y cerdos que genera unas considerables pérdidas económicas y discapacidades. La transmisión del parásito está vinculada a factores ambientales y de comportamiento como un saneamiento y higiene inadecuados, el mal manejo del cerdo y el consumo de carne de cerdo infectada. Este método de investigación aplicó un método de investigación para diseñar una estrategia de intervención de educación sanitaria con el fin de reducir las infecciones por T. solium en Burkina Faso, un país endémico para este parásito.
Métodos: Se llevaron a cabo dieciocho reuniones de grupos formados por 8 - 18 participantes en cada una de las tres aldeas. Además se realizaron entrevistas estructuradas entre los 4.777 participantes y 2.244 propietarios de cerdos, que fueron seleccionados mediante muestreo aleatorio de grupos en 60 aldeas de tres provincias de Burkina Faso. Con ambos enfoques se aplicaron conocimientos y prácticas relacionadas con T. solium. La información obtenida se utilizó para desarrollar una estrategia de intervención de educación sanitaria adaptada a cada comunidad para el control de la teniasis y la cisticercosis en Burkina Faso.
Resultados: Las reuniones de los grupos revelaron que los participantes tenían una mala calidad de vida debido a las enfermedades, así como el insuficiente acceso a letrinas, agua potable y servicios sanitarios. Además se averiguó que la producción de cerdos era una actividad económica importante, especialmente para las mujeres. Por otra parte, la escasa capacidad financiera y de conocimientos eran importantes limitaciones para una mejor gestión de los cerdos y de la construcción de letrinas. Los datos del estudio también demostraron que la defecación al aire libre y el agua sin hervir utilizada para beber eran comportamientos habituales, así como la falta de conocimiento sobre la transmisión del parásito, las barreras financieras percibidas como un obstáculo para la implementación de medidas de control, la falta de sensibilización pública, así como la falta de autoeficacia para el control del parásito. Sin embargo, las prestaciones económicas percibidas para el control de la cisticercosis porcina se podrían mejorar con un programa educativo que desalienta la defecación al aire libre y anime a beber agua potable en buenas condiciones. La estrategia de intervención final incluyó la elaboración de un plan sanitario y de higiene denominado PHAST (Participatory Hygiene and Sanitation Transformation), así como una película de 52 minutos y un folleto adjunto con viñetas cómicas.
Conclusiones: El principal problema en las comunidades estudiadas respecto a la transmisión de cisticercosis por T. solium es la disposición aleatoria de las heces humanas, que pueden estar contaminadas con huevos de parásitos. Evitar la defecación al aire libre exige construir letrinas, lo cual puede ser bastante problemático en entornos con dificultades económicas. Proporcionar a la comunidad los conocimientos necesarios para construir letrinas duraderas utilizando materiales de bajo coste disponibles en la zona puede ayudar a resolver este problema. Es preciso realizar más

estudios para implementar y evaluar la estrategia de control de T. solium desarrollada en este estudio.

Translated from English version into Spanish by SergioLorenzi, through

TRANSLATORS
WITHOUT BORDERS
