# Supplementary material for: Development of a health education intervention strategy using an implementation research method to control taeniasis and cysticercosis in Burkina Faso
Source: Infect Dis Poverty. 2017 Jun 1;6:95. doi: 10.1186/s40249-017-0308-0 (PMC5452375; doi:10.1186/s40249-017-0308-0)
Supplement: Supplementary file 2 — A questionnaire to assess participants’ knowledge and practices related to Taenia solium cysticercosis and taeniasis in 60 villages in Burkina Faso between Feburary 2011 and January 2012. (DOC 49 kb) [file 40249_2017_308_MOESM2_ESM.doc]

##### ÉFÉ CAB

***Improving pig management to prevent epilepsy in Burkina Faso***

Centre Hospitalier Universitaire Souro Sanou, AFRICSanté & University of Oklahoma Health Sciences Center

# SCREENING QUESTIONNAIRE

Last name : _______________________ First name : _____________________________

Questionnaire number _____________________

Identification number |__|__|__|__|__|__|__|__|__|__|__|__|

Village __________________________________

Concession __________________________________

Household number _______________________

How long have you lived in this village?____(yrs.)

Do you benefit from a health insurance?🞎 Yes 🞎 No

If yes, what type of health insurance is it?

🞎Private Health insurance 🞎Mutual Health insurance 🞎Other[*specify*] ________

1 How old are you? _____________ (years)

2 What is your date of birth?____ Day _____ Month _______ Year

3 Sex 🞎 Male 🞎 Female

4.Did you go to a modern school? 🞎 Yes 🞎 No [*please go to Q6*]

5. What is the last grade you attended? __________________________

5.1 What is the highest schooling grade you have completed?

🞎 None 🞎 CEPE 🞎 BEPC 🞎 BEP/CAP

🞎 Baccalauréat 🞎 University degree

6 What is your usual occupation, in other word, what work do you do most of the time [*housewife is an occupation*]?

🞎 Farmer 🞎 Small business 🞎 Handicraft

🞎Salaried (specify) _______________ 🞎 Housewife

🞎 Other (specify) _____________________

6.1 If you have a usual occupation, what is your monthly salary? __________________ CFA

7How many days of work have you missed because of illness in the past month? ______ days

7.1 If you do not have an official employment, how many days have you been unable to attend to your daily chores in the past month (past 30 days)? _____ days

7.2 What illness was it? ______________________________________________

7.3 Did you have diarrhea (at least three episodes of liquid or loose stools in one day) during the past two weeks?🞎Yes 🞎 No

8 How many days of work have you missed because of illness in the past year (past 12 months)? ______ days

8.1 If you do not have an official employment, how many days have you been unable to attend to your daily chores in the past year (past 12 months)? _____ days

8.2 What illness(es) was it? ______________________________________________

9 Where do you usually get your drinking water?

🞎Tap water 🞎Open well 🞎Traditional well

🞎Drilled well 🞎Spring 🞎 River / pool 🞎Other (specify) _____________

10Do you boil your drinking water?

🞎 Always 🞎Almost always

🞎 Sometimes 🞎Never

11Do you eat pork meat? 🞎 Yes 🞎 No [*Skip to Q11.a then Q13*]

11.a If no, did you use to eat pork meat? 🞎 Yes 🞎 No

11.1How often do you eat pork?

🞎 At least once a month 🞎 Less than once a month but at least once a year

🞎 Less than once a year

12.1How is the pork that you eat prepared? [*Check all that apply.*]

🞎 Boiling 🞎 Barbeque

🞎 Fried 🞎 Others [*Specify*]______________________________

12.2 Have you ever eaten [*Check all that apply.*]

🞎 Raw pork meat 🞎Rare pork meat

🞎 Medium cooked pork meat 🞎Well done pork meat

🞎 Cannot remember, do not know

12.3 Where do you usually eat pork meat [*Check all that applies*]

🞎At home 🞎At another concession in the village

🞎 At the village’s market 🞎At another village’s market

🞎 Other (specify) ______________________________________________

13 Do use a latrine?

🞎1 Yes 🞎2 No [*Skip to Q14*]

13.1 How often do you use a toilet when you have to defecate?

🞎1 Always 🞎2 Sometimes 🞎3 Never

15 Have you ever owned pigs (now or in the past)? *[If they answer “yes”, read options 1, 2 and 3]*

🞎1 Yes, in the past 12 months 🞎2 Yes, one (1) to five (5) years ago

🞎3 Yes, more than five (5) years ago

🞎4 No[*Skip to Q 17*]

16 Were you ever told that your pigs or piglets were infected with cysts (cysticercosis)?

🞎1 Yes 🞎2 No [*Skip to Q 17*]

- 1. When were you told that your pig or piglets were infected with cysts (cysticercosis)?

🞎1 In the past year 🞎2 One (1) to five (5) years ago

🞎3 More than five (5) years ago

🞎4 Never told (skip to Q 17) 🞎5Can not remember, do not know (Skip to Q17)

17 Have you ever seen or heard of white nodules (rice) in pig carcasses?

🞎1 Yes 🞎2 No [*Skip to Q 18*]

17.1 Where can you find nodules on a live pig?

🞎1 It is not possible to find them on a live pig

🞎2 Under the skin 🞎3 Under the tongue

🞎4 I don’t know 🞎5Somewhere else [*Specify*] ______________

17.2 How do pigs get these nodules?

🞎1 By eating human faeces 🞎2By eating pig faeces

🞎3 From another infected pig 🞎4 Other [*Specify*] ___________________

🞎5 I don’t know

17.3 How did you hear about those nodules in pigs?

🞎1 By a meat inspector 🞎2 By a pig trader

🞎3 BY a traditional healer 🞎4 At the radio / in the newspaper

🞎5 By a friend 🞎6 By ÉFÉCAB

🞎6 Other (spécify) ________________________________________________

18 Have you ever heard of tapeworm infection in humans?

🞎1 Yes 🞎2 No [*Skip to Q19*]

18.1 How did you learn about it?

🞎1 By a doctor 🞎2By a friend or family member

🞎3 By a traditional healer 🞎4On the radio / newspaper

🞎5 Other [*Specify*] ________________________________________________

18.2 How does a person know if they have a tapeworm?

🞎1 They can see it in their faeces 🞎2They have diarrhoea

🞎3 They have fever 🞎4 Other [*Specify*] ________________________

🞎5 I don’t know

18.3 Have you ever had a tapeworm or seen small parts (segments) of worms that look like rice grains in your faeces? (*Show photographs of proglottids*)

🞎1 Yes 🞎2 No [*Skipto Q 18.4*]

🞎3 I don’t know/can not remember [*Skipto Q 18.4*]

18.3.1 When that happened, what did you do? [*check all that applies*]

🞎1 Went to a primary health care provider (hospital, clinic, dispensary) 🞎2 Went to the pharmacy to get a drug to treat it

🞎3 Went to a traditional healer 🞎4Did nothing

🞎5 I can not remember, I do not know

18.4 How does a person get tapeworm infection?

🞎1 They do not wash their hands 🞎2They eat undercooked pig meat

🞎3 They are in contact with an infected person🞎4 Other [*Specify*] _________

🞎5 I don’t know

The remainder of the questions are not shown here as they had to do with screening of severe chronic headaches and epilepsy, and not measuring KAP.

**THIS IS THE END OF THE INTERVIEW**

## THANK YOU VERY MUCH FOR YOUR COOPERATION

INTERVIEWER: _______________________________________DATE OF INTERVIEW
